# Cervical and Oral Screening for HR-HPV types 16 and 18 among Sudanese Women Cervical Lesions

**DOI:** 10.1186/1750-9378-7-17

**Published:** 2012-07-31

**Authors:** Abdelbaset Mohamed Elasbali, Afra Hassan Saad El Din, Rania Abdeen Hussein Abdallah, Hussain Gadelkarim Ahmed

**Affiliations:** 1 Department of Histopathology and cytology, University of Khartoum, Khartoum, Sudan; 2 Department of Histopathology and cytology, FMLS, Sudan University for Science and Technology, Khartoum, Sudan; 3 Department of Gynecology and Obstetrics, Omdurman Hospital, Omdurman, Sudan

**Keywords:** HR -HPV 16, 18, Cervical lesions, Sudanese

## Abstract

**Objective:**

This study examined whether there is a positive correlation existed between cervical and oral High Risk-Human Papilloma Viruses (HR-HPV) types 16, 18 infections in patients with clinically confirmed cervical lesions.

**Methods:**

In this study 50 participants were included (40 were cases and 10 were controls). One hundred DNA materials (50 were cervical and 50 were oral epithelial tissues) were analyzed using HR-HPV subtypes 16 and 18 specific PCR probes.

**Results:**

Of the 40 cases, HR-HPV 16, 18 were identified in 16/40 (40%), of the cervical tissues of whom 8/16 (50%) were positive for HPV 16; 6/16 (37.5%) were identified with HR-HPV 18, and 2/16 (12.5%) were detected with both HR-HPV subtypes. All of the clinically healthy cases were found negative. Only one oral tissue sample (case) was 1/40 (2.5%) was found positive for HPV subtype16.

**Conclusion:**

The frequency of infection with HR-HPV subtypes 16 and 18 is high among Sudanese women with cervical lesions and suggests a role of HR-HPV in the development of cervical cancer in Sudan. No correlation between cervical and oral HPV infection was noted. Further study with screening of large number of patients with cervical cancer is recommended for further clarification of these findings.

## Introduction

Cervical cancer is the second most common cancer found in women with approximately 530,000 new cases each year resulting in an estimated 275,000 deaths, worldwide. In developed countries, cervical cancer incidences have declined, mostly due to cervical cytology screening campaigns, which requires significant medical resources and laboratory infrastructure. Cervical cancer is on the rise in the developing world, with one-seventh of the world’s cervical cancer cases in China, where no nationwide screening program for the disease currently exists [1]. In Sudan, cervical cancer is the second most common cancer type among women [2] and there are 923 new cases representing 4.5/100,000 [3]. The researchers have proposed that Human Papilloma Viruses (HPV) testing of self-collected Pap specimens might serve as an alternative or complementary method of a primary cervical cancer screening method [1]. As long ago as 1995 the causative association between HPV and Squamous Cell Carcinoma (SCC) was recognized [4]. Epidemiological studies demonstrated that the major risk factor for the development of pre-invasive and invasive carcinoma of the cervix is HPV infection. In an international study consisting of 1000 specimens from patients with invasive cancer in 32 hospitals in 22 countries ,HPV-DNA was present in 99.7% of cervical cancers, HPV16 was the predominant type in all countries except Indonesia where type 18 was more common [5]. HPVs are small, non-enveloped DNA viruses. HPV infects and replicate within cutaneous and mucosal epithelial tissues. The HPV family of viruses contains more than 100 types. Approximately 40 HPV types affect the genital area. They can be sub-divided into low risk for cervical cancer and high risk [6]. The high risk are associated with the development of cervical cancer includes (types 16, 18, 31, 33, 35, 39, 45, 51, 52, 56, 58, 59, 68, 73, 82) [7]. The low-risk types (HPV 6, 11, 40, 42, 43, 44, 54, 61, 70, 72, 81, and CP6108) can cause mild cervical dysplasia but are rarely associated with severe cervical dysplasia or cervical carcinoma [8,9].

HPV infection has also been postulated as a potential risk factor for OSCC. Several studies have detected HPV DNA in a considerable proportion of oral cancers, with wide variations from 0% to 100% prevalence in oral tissues, perhaps reflecting the inherent variations in the different populations, [10-12].

In Sudan, invasive cervical cancer is a leading cause of cancer death among women [13] . Most cervical cancers are squamous cell carcinomas representing 90.9%, followed by adenocarcinomas 4.8%, and other epithelial tumors were 4.3%. Of the squamous carcinomas, 98.8% were invasive and 1.2% intraepithelial (cervical intraepithelial neoplasia). The majority of cases presenting with a protruding cervical mass [14]. Although there are many suggested risk factors for cervical cancer [15], HPV remains the prime suspect.

Furthermore, a recent study has reported strong association between HPV types 16 and 18 and OSCC, although, some prior studies have reported a lack [16] or low association between HPV and OSCC among Sudanese patients [17].

However, due to the lack of published data from Sudan in this context, the aim of this study is to examine whether there is a positive correlation existed between cervical and oral HR-HPV types 16, 18 infections in patients with clinically confirmed cervical lesions.

## Materials and methods

### Study design

In this descriptive study, 40 patients with clinically confirmed cervical lesions (cases) 10 clinically confirmed with no cervical lesions (controls) were investigated for the presence of HR-HPV subtypes 16 and 18 PCR using molecular techniques. All subjects were randomly selected from those referred to gynecologic clinic at Al Nou hospital. All Participants were with apparently healthy oral cavity.

### Ethical consent

The study was approved by the ethics board of the Faculty of Medical Laboratory Science, Sudan University for Science and Technology. All cervical samples were taken as a part of the specimens required for investigation. All study subjects were consented to participation by completing the self-administered questionnaire.

### Sample processing

The sample size represents a full coverage of patients that were referred to the gynecologic clinic and agreed to participate within 6 months period. One hundred samples (50 cervical scrapes and 50 oral scrapes) were collected as a part of the specimen required for screening for cervical cancer. Both cervical and oral cells were obtained from the same person and processed similarly. B scraped cells placed in sterile Caryo-tube containing 5 ml Tris–HCl buffer (PH 8.0) (preparation stock A 2.42 gm Tris in 100 cm3 of distill water, stock B 1.7 cm3 hydrochloric acid in 100 cm3 distill water, 25 cm3 of A + 13.4 of B made up to 100 cm3 with distill water). The components were then centrifugated and the supernatant was discarded, and the deposit was kept immediately in the refrigerator in −200 C.

### DNA extraction

DNA was extracted according to the steps described in DNA extraction kit purchased from Sacace biotechnologies-Casera –Italy. The pellet obtained from previous steps was treated with 300 μl of Reagent 2 (lysis buffer) in addition 100 μl of sample, vortexed, incubated at 65°C for 5 min and centrifuged at (12000–16000 g) for 10 min and transfer the supernatant into new tube (sterile 1.5 ml Eppendorf tube) for DNA extraction . Vortexed vigorously sorbent and added 20 μl to each tube, Vortexed for 5–7 sec and incubated all tubes for 3 min at room temperature, then this step was repeated. Then all tubes were centrifugated for 30 sec at 5000 g and used amicropipette with aplugged aerosol barrier tip, carefully removed and discarded supernatant from each tube without disturbing the pellet. Tips was Changed between the tubes. 500 μl of Washing Solution was added to each tube. Vortexed vigorously and centrifuged for 30 sec at 10000 g. Supernatant was removed and discarded from each tube. This step was repeated and incubated all tubes with open cap for 5–10 min at 65°C. The pellet was resuspended in 100 μl of DNA-eluent. Incubate for 5 min at 65°C and vortex periodically. The tubes were centrifuged for 1 min at 12000x g. The supernatant was containing DNA ready for amplification stored at - 20°C until used.

### Polymerase chain reaction (PCR) Amplification of HPV

Type specific primers (primer for HPV 16 and HPV18) were used to detect HPV16 and 18 DNA in oral benign and malignant lesion. Amplification was performed according to HPV16/18 kit from Sacace-Biotechnologies S.r.l. Caserta –Italy. The final reaction volume of 40 μl containing 20 μl mix-1 (contained in PCR tubes), 10 μl of mix-2 and 10 μl of extracted DNA (sample). Negative control, positive HPV16 DNA and positive control 18 DNA tubes contained 10 μl of DNA buffer, 10 μl of HPV 16 DNA and10μl of HPV18 DNA respectively. Samples and controls were amplified using Gene Amp PCR system 9700. The PCR program is described in Table 1.

**Table 1:** PCR steps

**Cycles**	**Time**	**Tem**	**PCR steps**
Pause		95°C	0
1	5 min	95°C	1
42	15 sec	95°C	2
	25 sec	65°C	
	25 sec	72°C	
1	1 min	72°C	3
**Storage**		4°C	4

### Gel-electrophoresis

The PCR products were visualized in 2% Agarose gel with 0.5 μg/ml Ethidium bromide. Ten micro liters of 100 bp DNA ladder and PCR product was loaded on the gel. Gel-electrophoresis was performed at 120 V and 36 mA for 60 minutes. Pictures were taken by Gel documentation system (Gel mega, digital camera and software in a computer).

### Interpretation of PCR results

According to manufacture HPV16/18 kit (Sacace-Biotechnologies S.r.l. Caserta –Italy) manual, the PCR product length for HPV16 should be 325 bp and 425 bp for HPV18.

### Data analysis

Data management was done by using the Statistical Package for Social Sciences (SPSS version 12; SPSS Inc, Chicago, IL). SPSS was used for analysis and to perform Fisher exact test for statistical significance (P value < 0.05 was considered significant). The 95% confidence level and confidence intervals were used.

## Results

**Figure 1:**
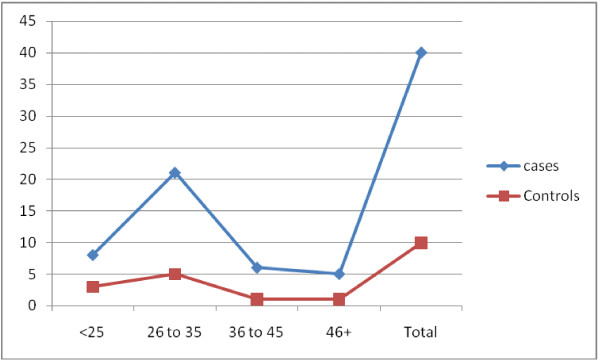
Description of cases and controls by age 1.

A total of 40 cases (patients with clinically confirmed cervical lesions) and 10 controls (patients without cervical lesions) were included in this study. The age distribution is relatively similar between cases and controls with a range of 18 to 70 years and a mean age of 33 years. Most of cases were middle age 26–35 years representing 21/40 (52.5%) followed by 18–25 group 8/40 (20%), as indicated in Table 2, Figure 1. The distribution of the study population by resident is shown in Table 1. Most of cases were from North followed by West and Centre constituting 13/40 (32.5%), 12/40 (30%), 12/40 (30%), respectively.

**Table 2:** Distribution of study population by age and residence

**Variable**	**Category**	**Cases**		**Controls**		**Total**
		**No**	**%**	**No**	**%**	
**Age**	18-25	8	20	3	30	11
	26-35	21	52.5	5	50	26
	36-45	6	15	1	10	7
	46-70	5	12.5	1	10	6
	Total	40	100	10	100	50
**Resident**	North	13	32.5	4	40	17
	West	12	30	2	20	14
	East	3	7.5	0	0	3
	Center	12	30	4	40	16
	Total	40	100	10	100	50

**Figure 2:**
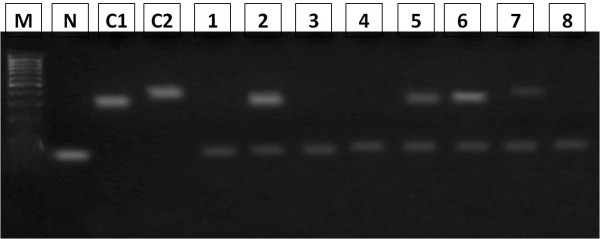
**Photomicrograph 1.** Ethidium bromide stained agarose gel 2% electrophoresis of HPV PCR products, carried out on DNA samples extracted from fresh cells , Lane M:100 bp ladder, (Arrows shows 300 and 400 band), Lane N negative control, Lane C1 positive control for HPV16, Lane C2 positive control for HPV18,Lane 1-2-3-4-5-6-7-8 extracted DNA samples.

**Figure 3:**
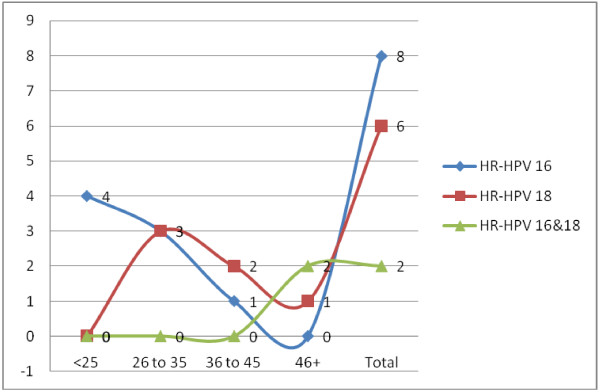
Description of the cases by HR-HPV infection.

**Figure 4:**
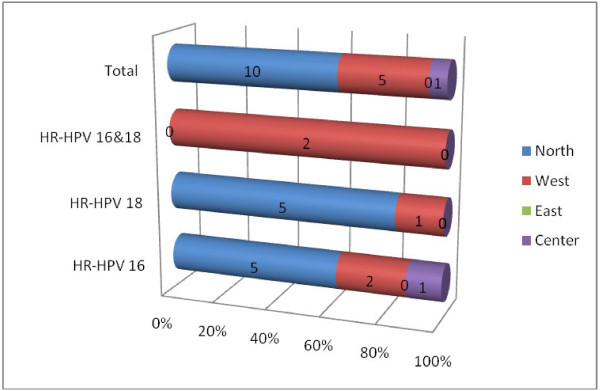
Description of the HR-HPV subtypes 16 and 18 by geographical distribution.

Table 2 summarizes the results of High Risk (HR-HPV) with other demographical factors. Of the 40 cases, HR-HPV 16, 18 were identified in 16/40 (40%), of the cervical tissues of whom 8/16 (50%) were positive for HPV 16; 6/16 (37.5%) were identified with HR-HPV 18, and 2/16 (12.5%) were detected with both HR-HPV subtypes, as indicated in Table 2, Photomicrograph1 (Figure 2). All of the clinically healthy cases were found negative. Only one oral tissue sample (case) out of 40 (1/40 = 2.5%) was found positive for HPV subtype 16. According to age, a high rate of infection with HR-HPV 16 was observed in among age group 18–26 years representing 4/8 (50%) followed by age range 26–35 representing 3/8 (37.5%). For infection with HR-HPV 18, the highest frequency was detected among age group 3/6 (50%) followed by age range 36–45 years constituting 2/6 (33.3%). The 2/2 (100%) cases infected with both HR-HPV 16&18 were found among age range 46–70 years, as shown in Table 3, Figure 3. According to the residence high frequencies of infection with HR-HPV 16 were found among patients from Northern Sudan representing 5/8 (62.5%) followed by Western Sudan 2/8 (25%), similarly, high frequencies of infection with HR-HPV 18 were seen among patients from Northern Sudan representing 5/6 (83.3%). Infections with both HR-HPV 16 & 18 were detected in two patients from Western Sudan, as seen in Table 3, Figure 4.

**Table 3:** Distribution of cases according to HPV DNA detection in cervical cells

**Variable**	**category**	**HR-HPV 16**		**HR-HPV 18**		**HR-HPV 16&18**	
		**+ve**	**-ve**	**+ve**	**-ve**	**+ve**	**-ve**
**Age**	18-25	4	4	0	8	0	8
	26-35	3	18	3	18	0	21
	36-45	1	5	2	4	0	6
	46-70	0	5	1	4	2	5
	Total	8	32	6	34	2	40
**Resident**	North	5	8	5	8	0	13
	West	2	10	1	11	2	10
	East	0	3	0	3	0	3
	Center	1	11	0	12	0	12
	Total	8	32	6	34	2	38

## Discussion

Worldwide, after breast cancer, cervical cancer is the second most common cancer that affects women. In 99.7% of all cases, cervical cancer results from a history of persistent infection by HR-HPV [5]. The high risk types occurring most frequently in cervical cancer include HPV-16 and HPV-18; together these account for over 70% of SCCs [18]. HPV-18 is also thought to account for approximately 50% of all adenocarcinomas [19]. Therefore, the present study mainly screened cervical and oral cells for the presence of these two HR-HPV subset.

HPV infection rates vary greatly between geographic regions and population groups [20,21]. In the present study, HR-HPV 16 and 18 were identified in 40% of the cervical specimens. In a meta-analysis which was performed on studies published between 1995 and 2009 that used polymerase chain reaction or Hybrid Capture 2 for HPV detection in women with normal cytological findings. The analysis included 194 studies comprising 1,016,719 women with normal cytological findings. The estimated global HPV prevalence was 11.7% (95% confidence interval, 11.6%-11.7%). Sub-Saharan Africa (24.0%), Eastern Europe (21.4%), and Latin America (16.1%) showed the highest prevalences. Among the women with type-specific HPV data (n = 215,568), the 5 most common types worldwide were HPV-16 (3.2%), HPV-18 (1.4%), HPV-52 (0.9%), HPV-31 (0.8%), and HPV-58 (0.7%) [22]. Prevalence of cervical HPV infection in most African countries is very high. HPV was detected in 100% of cervical biopsies from 70 women with cervical cancer in Papua New Guinea, with HPV types 16 and 18 being the most prevalent at 57.1% and 25.7% (95% CI, 0.45-0.68 and 0.17-0.37) respectively [23]. High frequencies of HR-HPV 18 and 16 were reported from Egypt [24], Morocco [25], 59%, 56% respectively. The only one study from Sudan, has reported 60.7% ß. globin positive samples for HPV indicating DNA integrity [26]. The authors analyzed HPV by general primer GP5+/6+ mediated PCR enzyme immunoassay (EIA) as described by Jacobs et al. [27] that its used to detect a broad spectrum of human papilloma virus (HPV) genotypes including the high-risk groups (HPV-16,18,31,33,35,39,45,51,52,56,58,59,66, 68 and 73) and low risk groups (HPV-6,11,40,42,43 and 44). Interestingly, the most common high risk infection in their study group was type 58, and in the low risk type 42.

In regard to age, most of positive cases were relatively observed among younger women, and these findings were consistent with global reports [28]. However, a meta-analysis have reported age-specific HPV distribution presented with a first peak at younger ages (< 25 years) and, in the Americas and Africa, a rebound at older ages (≥ 45 years) [22].

According to the residence, most of cases were from Northern Sudan. However, this might be due to the fact that Northern Sudan consist the most civilized part of the Sudan, this in addition to the fact the study was performed in the Northern Sudan.

In this study, oral HPV infection was found to be very low prevalent (2.5%) than cervical HPV infection (40%). Most women with an oral HPV infection also had a cervical infection, likely because the majority of women had a cervical infection. The increased prevalence of oral infections in women with simultaneous cervical infections would suggest that the behaviors that place a woman at risk for oral HPV infection could substantially overlap with cervical infection. Anogenital HPV infections in adults are predominantly sexually transmitted [29], and indeed, sexual behaviors have been previously associated with oral HPV infection [30]. Concomitant type-specific infections at both anatomic sites could be obtained during the same sexual encounter or sequential sexual encounters with an infected partner or autoinoculation from one site to the other [31]. However, only one study from the Sudan in this context has evaluated the possible role of high risk Human Papilloma viruses (HPV) 16 and 18 in oral squamous cell carcinomas (OSCC). HPVDNA was detected in 15% of cases (six out of 40 cases), and none of controls (n = 15), P < 0.0001 [32].

## Conclusion

HR-HPV types 16 and 18 are the most commonly detected in Sudan and relatively similar to those described in neighboring African countries, although the relative contribution of HPV16 and HPV18 is substantially lower in cytologically normal women. The oral and cervical infection for HPV infection are likely independent of one another. Further more comprehensive studies are recommended for stronger clarification of association between HPV infection and cervical cancer in Sudan.

## Competing interests

The authors declare that they have no competing interests.

## Authors’ contributions

AME: involved in the practical work (PCR). AHSED: involved in the practical work (PCR). RAHA: Patients management. HGA: consultation and Manuscript preparation. All authors read and approved the final manuscript.
